# Could Spinal Epidural Lipomatosis Be the Hallmark of Metabolic Syndrome on the Spine? A Literature Review with Emphasis on Etiology

**DOI:** 10.3390/diagnostics13020322

**Published:** 2023-01-16

**Authors:** Valerio D’Agostino, Miriana Rosaria Petrera, Giuseppe Tedesco, Valerio Pipola, Federico Ponti, Paolo Spinnato

**Affiliations:** 1 Diagnostic and Interventional Radiology, IRCCS Istituto Ortopedico Rizzoli, 40136 Bologna, Italy; 2 Spine Surgery, IRCCS Istituto Ortopedico Rizzoli, 40136 Bologna, Italy

**Keywords:** magnetic resonance imaging, obesity, visceral adipose tissue, non-alcoholic fatty liver disease, spinal stenosis, multidetector computed tomography

## Abstract

Spinal epidural lipomatosis is defined by an excessive amount of epidural fat in the spinal canal, usually in the lumbosacral tract: a well-known cause of lumbar pain and spinal stenosis with a possible wide range of neurological symptoms. Recent research data reveal that, nowadays, obesity has become the main cause of spinal epidural lipomatosis. Moreover, this condition was recently recognized as a previously unknown manifestation of metabolic syndrome. Radiological studies (CT and MRI) are the only tools that are able to diagnose the disease non-invasively. Indeed, radiologists play a key role in disease recognition, with subsequent possible implications on patients’ systemic health assessments. Despite its clinical importance, the condition is still underreported and neglected. The current literature review summarizes all the main etiologies of spinal epidural lipomatosis, particularly regarding its linkage with metabolic syndrome. An overview of disease characteristics from diagnosis to treatment strategies is also provided.

## 1. Introduction

Spinal epidural lipomatosis (SEL) is a condition characterized by the over-deposition of unencapsulated epidural fat in the spinal canal, usually in the lumbosacral tract. The non-invasive diagnosis of SEL can be made with Magnetic Resonance Imaging (MRI) or Computed Tomography (CT) only.

SEL, a pathologic condition, may lead to the narrowing of the spinal canal and compression of surrounding neural structures. SEL can be asymptomatic in mild–moderate disease and usually becomes symptomatic in moderate–severe cases. The clinical presentation typically involves progressive lower back pain radiating to the lower limbs, spinal claudication, radiculopathy, myelopathy, and cauda equine syndrome [1,2].

In most cases, symptoms develop over months to years but may develop acutely in some rare cases [3,4,5,6]. For unclear reasons, males are affected more commonly than females (M:F = 3:1) [7]. Moreover, more than 75% of all reported patients are obese [8]. Exceptionally, children can suffer from neurological symptoms associated with SEL, and this is often accompanied with a long history of steroid treatment [9,10,11,12].

A previous large population study based on MRI reports including 28,902 subjects reported a prevalence of SEL of approximately 2.5%. In this series, the main factors associated with the disease included being male, obese, and having a history of systemic corticosteroid use [13]. Recent work has focused attention on the ratio of radiologists’ awareness and the detection of this condition, highlighting a fairly significant underestimation of SEL in radiologists’ reports and suggesting that the actual prevalence of SEL has been underestimated so far [14]. The prevalence of SEL in this latter research study, which includes 450 subjects, was notably higher (16.7%) [14]. Another previous large series focused on lumbar MRIs and including 2528 patients found quite similar results (SEL prevalence = 20.8%) [15].

Historically, steroid excess (exogenous or endogenous) was considered the most common etiological factor associated with SEL [16,17,18]. Indeed, the first case of steroid-induced lipomatosis was reported by Lee et al. in 1975 [19].

Because obesity in adults is usually accompanied by common non-communicable diseases, such as dyslipidemia, Type 2 diabetes, hypertension, and arteriosclerosis, the high rate of obesity in patients with idiopathic SEL suggests that non-communicable diseases and SEL are causally related. The primary line of treatment is to reduce or eliminate the etiologic factors. When a clear etiologic factor cannot be ruled out, weight loss has been proposed as a conservative treatment. However, surgical decompression is the treatment of choice, especially for acute or severe cases [20,21]. Moreover, in patients with worsening or persistent neurologic symptoms, surgery should be suggested [1,21,22].

When SEL is a concause of neurological symptoms, together with other conditions (i.e., thickened yellow ligament and disc herniation), a negative prognostic impact on the surgical outcome of posterior decompression surgery has been reported [23], giving paramount importance to its correct reporting by radiologists.

Other reported secondary causes include adrenal tumors, hypothyroidism, hyperprolactinemia, and other endocrinopathies. Nevertheless, several cases of SEL are considered idiopathic because of the absence of clear predisposing factors. Our work aims to thoroughly research the literature-based evidence of the correlation of SEL with miscellaneous causes and emphasizes its link with metabolic syndrome.

## 2. SEL’s Etiology

In the following subsections, the recognized causes of SEL are reported and discussed, with reference to the available literature.

### 2.1. Excessive Amount of Corticosteroids

SEL has been frequently associated with excessive use of corticosteroids, which can have an exogenous source-like steroid injection [24] or long-term corticosteroid therapy [25,26,27,28]. It is also associated with an endogenous source-like corticotropin syndrome from ACTH-secreting extra pituitary tumors (endogenous Cushing’s syndrome) [17] or from an ACTH-secreting pituitary gland adenoma (Cushing’s disease) [29,30].

Among the series that have been reported, approximately 75% of cases have been associated with exogenous steroid use [31], with great variability from case to case in terms of the doses and duration of treatments associated with this condition [32]. Mostly, moderate-to-high steroid dosages were received by patients for years before the development of symptoms.

Although relatively rare, some cases of patients who developed SEL with a long-term and low-dosage steroid treatment have been described in the literature [33].

When the treatment with steroids is both long-term and high-dosage, there is a risk of developing severe SEL, even in children, as evident in an 8-year-old patient in treatment for Crohn’s disease who suffered from myelopathy due to the SEL-induced compression of the spinal cord [34].

There have been reports of SEL associated with epidural steroid injections and even inhaled steroids, but the long-term use of oral steroids is by far the most common association [35,36,37].

Koch et al. described a case of a patient with bronchial carcinoid associated with ectopic corticotropin syndrome. The patient complained of severe back pain and leg weakness among other clinical issues. The MRI showed multiple levels of excessive epidural fat, which reverted to normal after two months of treatment with the inhibitors of steroidogenesis (metyrapone and ketoconazole). At the same time, there was a complete resolution of neurological symptoms [38].

Bathia et al. reported a similar case of ACTH-secreting bronchial carcinoid in a male farmer with Type 2 diabetes mellitus. The MRI showed multiple levels of SEL of the thoracic spine, without significant disc herniation. Since the patient’s pain was improving, despite treatment with ketoconazole, he was successfully treated with decompressive hemilaminectomy and the removal of the epidural adipose tissue (no stabilization was performed due to massive osteoporosis) [39].

A 17 yo with an ACTH-secreting adenoma of the pituitary gland, which developed after trans-sphenoidal adenectomy, experienced a sudden spastic paraparesis with a sensory deficit to the level of T5. An MRI scan showed a severe SEL of the thoracic spine, which was treated conservatively with success. This case shows that it is important to rule out SEL from a myopathy caused by hypercortisolism, starting with a proper collection of the patient’s clinical history [40].

Historically, SEL was associated with exogenous steroid use as the first cause of disease, even if recent reports suggest that obesity is becoming the major risk factor. A study by Fogel et al. reports that 55.8% of SEL cases caused by exogenous steroids affect the thoracic spine, compared to 32.7% that only involve the lumbosacral region and 11.5% that affect both [2]. This differs from the endogenous steroid disease-related SEL, which affects the thoracic and lumbosacral areas relatively the same. In addition, most obesity-related SEL (69.6%) and idiopathic-related SEL (50%) result in lumbosacral involvement.

On the other hand, a recent systematic review with meta-analyses revealed that obesity is the current leading cause of SEL [41].

### 2.2. SEL and Obesity

Obesity has always been considered one of the most common causes of SEL [2]. Nonetheless, in recent years, obesity is believed to be the most common cause of SEL. A recent meta-analysis confirmed that obesity should be considered the main risk factor for SEL development (52% of cases) [41].

Consistent with previous studies, there was a positive correlation between the degree of obesity and the severity of SEL [42,43,44,45].

In a large series by Theyskens et al., which included MRI scans of 28,902 patients, a BMI > 30 was independently associated with SEL in a multivariate analysis [13]. Yildirim et al. reported the strong correlation of an increased BMI with SEL in a retrospective case-matched control study including 199 patients. In this research study, the authors indicated that patients with SEL had a significantly higher median BMI than control subjects (36.7 vs. 29.4 kg/m^2^, *p* < 0.001) [46].

### 2.3. Non-Alcoholic Fatty Liver Disease (NAFLD)

Non-alcoholic fatty liver disease (NAFLD) is nowadays the most common chronic liver disease in developed countries. The clinical presentation of NAFLD ranges from asymptomatic (only with elevated liver enzyme levels) to cirrhosis, with possible complications of liver failure and hepatocellular carcinoma. There is sufficient evidence supporting an association between NAFLD and metabolic syndrome [47]; interestingly, recent studies have also found an association between SEL and NAFLD [48].

### 2.4. Miscellaneous

Other possible risk factors for SEL development have been suggested in recent years, but their role remains controversial and needs further investigation.

The principal difficulty in defining their real role in the development of SEL is the contemporary presence of well-known risk factors such as obesity and exogenous steroid treatment. More studies are needed to assess if they can be addressed as risk factors or only associated factors.

SEL has been described as a possible manifestation of highly active antiretroviral therapy (HAART)-associated lipodystrophy in HIV-positive patients [49,50,51,52].

Androgen deprivation therapy for prostate cancer has been suggested as a possible risk factor for SEL in several case reports [53,54,55,56].

Three case reports have described an association between SEL and scoliosis [57,58,59]. The rapid progression of SEL has been reported after spinal surgery [4,5,60].

In the study by Okada et al., SEL was significantly associated with diffuse idiopathic skeletal hyperostosis (relative risk (RR), 2.6; 95% confidential interval (CI), 1.3–5.1; *p* < 0.01) [61].

Finally, some authors have suggested a possible association between SEL and spinal kyphotic deformities, such as congenital, Scheuermann, and tuberculotic kyphosis [62,63]; Paget disease [64,65]; hypothyroidism [66]; chronic alcoholism [67]; and Type 1 diabetes mellitus [68].

### 2.5. Idiopathic

Idiopathic cases of SEL have been reported in the literature since 1982 [69]. Haddad et al. first hypothesized that idiopathic SEL was a byproduct of obesity, with the gradual overgrowth of epidural fat resulting in the compression of the spinal cord and nerves [70]. Nevertheless, cases of SEL in underweight patients suggest another kind of pathogenetic mechanism for idiopathic SEL [31].

The recognition of idiopathic SEL is of paramount importance, and in absence of risk factors, the disease cannot be clinically suspected. Patients may remain undiagnosed for a long period. As a result, the longer duration of nerve root constriction may cause a relatively lower recovery rate [71].

There is disagreement in the literature regarding the definition of idiopathic SEL. Indeed, some authors refer to idiopathic diseases to indicate the SEL of an unknown cause, while others use this term to describe SEL associated with obesity or other causes that nowadays are clearly risk factors for disease development. Indeed, the term “idiopathic SEL” should be used only in patients without any other recognized risk factor (e.g., metabolic syndrome, obesity, and increased corticosteroids).

In the literature review and meta-analysis by Fogel et al., idiopathic SEL has been reported to account for 17% of the cases. This group includes those patients who did not take exogenous steroids, were not obese, and did not have an underlying endogenous steroid hormonal disease [2,72,73].

Idiopathic SEL is also reported in the pediatric age, with few reports in the literature at this regard [74,75]. Importantly, the grading of SEL (including a cut-off for diagnosis) was tested only in adult patients, while pediatric-age patients’ range of normality in terms of amount of epidural fat is still missing [15]. Indeed, the diagnosis at this age is purely qualitative depending on the MRI or CT evaluation.

Racial differences may become more prevalent when discussing the pathogenesis of SEL.

Yoo et al. reported an incidence of up to 68.8% of idiopathic SEL in the Korean population [76], while Fogel et al. reported 17% for the same SEL category in Western countries [2].

This difference may suggest a role of genetic variation in the pathogenesis of SEL.

### 2.6. Epidemiological Analysis of SEL’s Etiologies

Historically, SEL was associated with exogenous steroid use as the first cause of disease [2], even if recent reports suggest that obesity is becoming the major risk factor [77]. A study by Fogel et al. reported that 55.8% of SEL cases caused by exogenous steroids affect the thoracic spine, compared to 32.7% that only involve the lumbosacral region and 11.5% that affect both [2]. This differs from the endogenous steroid disease-related SEL, which affects the thoracic and lumbosacral areas relatively the same. In addition, the majority of obesity-related SEL (69.6%) and idiopathic-related SEL (50%) result in lumbosacral involvement.

On the other hand, recently, obesity has been recognized as the main cause of SEL. Indeed, a recent systematic review and meta-analysis (time period considered 1990–2020) revealed that obesity is currently the main cause of SEL, accounting for more than a half of patients [77]. In Table 1 we summarized the main causes of SEL according to the latest systematic review and meta-analysis by Alomari et al. [77], compared with the data from a previous analog review and meta-analysis by Fogel et al. [2].

## 3. SEL and Metabolic Syndrome

According to the International Diabetes Federation [78], metabolic syndrome is defined as a condition with central obesity that is accompanied by any of the following disorders: hypertension (HT), raised triglycerides, reduced HDL cholesterol, and raised fasting plasma glucose.

These factors are associated with the onset of cardiovascular disorders and reduced life expectancy [79]. Metabolic syndrome is closely associated with visceral fat deposition rather than subcutaneous fat deposition [80]. Intra-abdominal fat volume measured from cross-sectional views at the level of the umbilicus has shown a good correlation with the visceral fat area in some studies [81]. Abe et al. evaluated the liver CT values in patients with SEL, which showed a strong positive correlation. Furthermore, multivariate analysis showed that the liver CT value was a significant explanatory variable for SEL, indicating that SEL may have a relationship with metabolic disorders, especially fatty liver. In addition, their study supported an association between NAFLD and metabolic syndrome [48].

**Figure 1:**
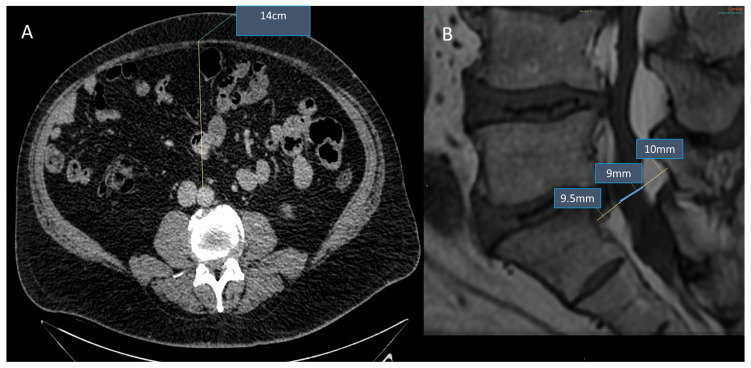
Obese patient (45 years old, male, BMI = 31 kg/m^2^, dyslipidemia). Association between SEL and visceral fat deposition: CT of the abdomen (Panel **A**) reveals increased visceral fat deposition (AP diameter 14 cm), while lumbosacral MRI sagittal T1w (Panel **B**) reveals SEL grade 2: moderate (dural-sac/epidural fat index = 0.46).

Ishihara et al. retrospectively reviewed 324 patients who underwent medical checkups and searched for a link between SEL and metabolic syndrome. In their study, SEL demonstrated a statistically significant correlation with BMI, abdominal circumference, and visceral fat deposition (Figure 1) [82].

They also divided subjects into two groups: those with a diagnosis of metabolic syndrome and controls. Interestingly, a logistic regression analysis of variables demonstrated that metabolic syndrome was significantly associated with SEL (Odds Ratio = 3.9; 95% CI = 1.5–9.8; adjusted for age, gender, smoking habits, and alcohol consumption) [82].

Besides subcutaneous and visceral fat, the concept of ectopic fat was recently proposed [83]. In humans, ectopic fat is found in the liver, skeletal muscle, pancreas, heart, and other organs. Metabolic syndrome leads to excessive visceral fat, and when it exceeds the normal limits, excess energy is passively stored in the liver and skeletal muscle, resulting in ectopic fat deposition [79].

The epidural space normally contains no more than a small amount of fat, and there is a possibility that the epidural fat in patients with SEL increases by a similar mechanism. Moreover, fat content in the liver and skeletal muscle is known to be related to insulin resistance, with a stronger correlation rather than with subcutaneous adipose tissue [84].

The accumulation of ectopic epidural fat in patients with SEL may be explained by similar pathological mechanisms such as metabolic syndrome and fatty liver.

Fujita et al. found a positive correlation between SEL and the “locomotive syndrome” which summarizes the physical impairment of a patient who underwent some clinical tests. The locomotive syndrome does not involve fat metabolism issues, but it has some condition overlapping with metabolic syndrome and risk factors as well (i.e., SEL); this could be another suggestion of SEL’s correlation with the latter [85]. 

In a study by Ishihara et al. including 166 male patients, severe SEL was found to be significantly associated with increased BMI as well as increased serum levels of total cholesterol and triglyceride [86].

Furthermore, the studies in the scientific literature discussed in previous paragraphs have proposed various risk factors for SEL; interestingly, many of them have a correlation and can be linked to metabolic syndrome (Table 2).

## 4. Diagnosis of SEL: The Role of Imaging

Radiological studies, namely CT and MRI, are currently the only medical tools able to diagnose SEL. The abnormal amount of epidural fat, most frequently found in the lumbosacral spine at the L5-S1 level, can be depicted on CT or even better with MRI, which offers an optimal characterization of adipose tissue, enabling the evaluation of neural roots and signs of stenosis.

Borrè et al. in 2003 proposed a 3-point grading system for the disease in its typical lumbosacral location, based on MRI measurements of epidural fat, the spinal canal, and the dural sac at L5-S1 level (Table 3) [15]. This grading system is still the standard reference for disease diagnosis and for grading SEL severity [93].

Currently, a grading system for SEL atypically located in the upper lumbar spine or in the thoracic tract is still missing.

The diagnosis of SEL may be incidental or clinically suspected based on clinical examinations and the presence of risk factors. Moreover, the disease is known to be always asymptomatic in Grade I, may be symptomatic in Grade II, and always symptomatic in Grade III [15]. It is worth mentioning that SEL can frequently be a concause for spinal stenosis symptoms, contributing to the narrowing of the central canal together with disk and facet joint diseases [14].

**Figure 2:**
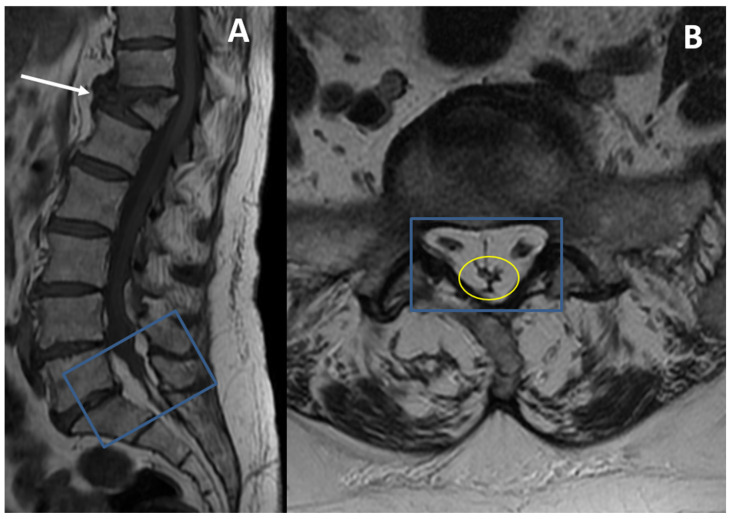
A 72-year-old woman affected by dyslipidemia, obesity, and high blood pressure underwent follow-up MRI control for a vertebral fracture (T12); patient complained about chronic lumbar pain, which worsened after the fracture, and bilateral sciatica. (**A**) Sagittal T1-weighted imaging (WI); (**B**) axial T1-WI. A marked vertebral body collapse of the T12 vertebra is detectable (white arrow). Moreover, a condition of SEL at L5-S1 level (blue rectangles) with compression of the thecal sac resembling the letter “Y” (yellow oval) is found.

In severe SEL (Grade 3), central canal stenosis may present a particular MRI appearance with the so-called “pseudo chord” appearance of neural roots (L1–L4 level) or the characteristics “Y sign” appearance of the thecal sac at L5-S1 level pathognomonic of this condition (Figure 2) [94,95]. The severe compression of thecal sac by hypertrophic epidural adipose tissue may also present a “stellar” appearance in the upper lumbar tract (usually L2–L4).

MRI permits the simultaneous assessment and grading of SEL and spinal cord damage/edema that can be present in thoracic locations of the disease.

The histopathologic findings of Geers et al. suggested that the morphologic and topographic features of the meningovertebral ligaments explain the polygonal, stellar, or Y-shaped deformation of the dural sac observed in axial CT and MR images in patients with lumbar epidural lipomatosis [96].

A comprehensive review of the literature about SEL performed by Reina et al. acknowledged that the type of cells and their distribution was fibrolipoma-like with a mixture of adipocytes and fibroblasts [97]. 

In this context, radiologists play a key role in the recognition of this disease. Indeed, radiologists should pay attention to epidural fat overgrowth, and they must know how to recognize it and grade its amount.

A recent large series by Spinnato et al. revealed that SEL reporting rate among radiologists’ MRI reports is still very low, accounting for only 8% of cases [14]. More attention should be paid to this possible diagnosis on cross-sectional imaging, both MRI and CT, considering the relevant related implication on neurological symptoms or pain, and on systemic health assessment.

## 5. SEL Treatment

The first line of treatment proposed for SEL is related to the etiologies of the disease. Indeed, the elimination of or reduction in the leading cause of SEL represents the first-line therapy [93]. Weight loss is a successful treatment for obesity-related SEL [98]. In the same way, a reduction in or suspension of steroid therapy is effective when this is the recognized cause of SEL. If conservative treatments fail in cases of acute/severe neurological symptoms, surgery is suggested with the decompression of the spinal canal [93]. Surgical full-endoscopic approaches have recently been reported to be successful at this aim [99]. Both uniportal and biportal approaches have been reported to be effective. In cases of extensive SEL with thoracic locations, minimally invasive hemilaminectomies has been successfully reported [100].

Moreover, recent studies highlighted the role of bariatric surgery as a treatment for obesity-related SEL, with proven efficacy with respect to the reduction in epidural fat amount together with a reduction in other possible co-existent extra-spinal lipomatosis (e.g., intra-abdominal and mediastinal) [101,102].

## 6. Conclusions

In conclusion, SEL may be considered a previously unrecognized manifestation of metabolic syndrome on the spine, and prevention could be prophylactic for SEL itself. At the same time, SEL detection on radiological exams (CT, MRI) can lead to an early diagnosis of metabolic disorders.

## Figures and Tables

**Table 1:** Data of SEL etiologies from a recent systematic literature review (time period 1990–2020) by Alomari et al. [77] and from a previous systematic literature review (time period 1975–2003) by Fogel et al. [2].

Literature Review (Time Period 1990–2020)—Causes of SEL	%
Obesity	52
Exogenous steroid use	22
Idiopathic	26
**Literature Review (Time Period 1975–2003)—Causes of SEL**	**%**
Exogenous steroid use	55.3
Obesity	24.5
Idiopathic	17
Endogenous steroid hormonal disease	3.2

**Table 2:** List of relevant articles in the literature, sorted by main risk factor attributed to SEL. Asterisks (*) indicate risk factors linked to metabolic syndrome.

Authors	Risk Factor	Number of Patients
Ishihara et al. [86]	Metabolic Syndrome (Obesity and Hyperlipidemia)	166
Yildirim et al. [46]	Metabolic Syndrome	199
Theyskens et al. [13]	Cushing’s Syndrome *	731
Bhatia et al. [39]	Cushing’s Syndrome *	1
Ahmad et al. [40]	Cushing’s Disease *	1
Ishihara et al. [82]	Hyperlipidemia *	324
Zhang et al. [41]	Obesity *	807
Cushnie et al. [87]	Obesity *	99
Walker et al. [88]	Obesity *	25
Fogel et al. [2]	Obesity *	104
Spinnato et al. [45]	Obesity *	1
Sasagasako et al. [89]	Subcutaneous Fat Thickness *	500
Lotan et al. [67]	Diabetes Type 1 *	3
Park et al. [90]	Diabetes Type 2 *	42
Morishita et al. [91]	Visceral Fat *	218
Abe et al. [48]	NAFLD *	102
Okada et al. [61]	DISH *	327
Khawaja et al. [7]	Hypertension *	2
Fasset et al. [16]	Exogenous Steroids	2
Tok et al. [92]	Epidural Steroid Injection	1
Billings et al. [49]	HAART-associated Lipodystrophy	1
Mattei et al. [53]	Androgen Deprivation Therapy	1
Papastefan et al. [9]	Idiopathic	1
Stern et al. [31]	Idiopathic	2

**Table 3:** MRI Grading System of Lumbar Spinal Epidural Lipomatosis proposed by Borrè et al. ref. [15]. EF = epidural fat, DuS = dural sac diameter, SC = spinal canal diameter.

SEL MRI Grading System			
MRI GRADE	DuS/EF Index	EF/SC Index (%)	Meaning
NORMAL (0)	≥1.5	≤40	Normal amount of EF
GRADE I	1.49–1	41–50	Mild overgrowth of EF
GRADE II	0.99–0.34	51–74	Moderate overgrowth of EF
GRADE III	≤0.33	≥75	Severe overgrowth of EF

## Data Availability

Not applicable.
